# Hyper-Heuristic Capacitance Array Method for Multi-Metal Wear Debris Detection

**DOI:** 10.3390/s19030515

**Published:** 2019-01-26

**Authors:** Yanshan Sun, Lecheng Jia, Zhoumo Zeng

**Affiliations:** 1 State Key Laboratory of Precision Measurement Technology and Instrument, Tianjin University, Tianjin 300072, China; sunyanshan@126.com (Y.S.); zhmzeng@tju.edu.cn (Z.Z.); 2 Beijing Hang Feng Ke Wei Equipment Technology CO., LTD., Beijing 100141, China

**Keywords:** wear debris, capacitance array sensor, hyper-heuristic PDE inversion, morphological extract characterization

## Abstract

Online detection of fatigued wear debris in the lubricants of aero-engines can provide warning of engine failure during flight, thus having great economic and social benefits. In this paper, we propose a capacitance array sensor and a hyper-heuristic partial differential equation (PDE) inversion method for detecting multiple micro-scale metal debris, combined with self-adaptive cellular genetic (SA-CGA) and morphological algorithms. Firstly, different from the traditional methods, which are limited in multi-induction-Dirac-boundary-inversion, a mathematical model with non-local boundary conditions is established. Furthermore, a hyper-heuristic method based on prior knowledge is also proposed to extract the wear character. Moreover, a 12-plate array circulating sensor and corresponding detection system are designed. The experimental results were compared with the optical microscopy. The results show that under the conditions of 1~3 wear debris with diameters of between 250–900 μm, the accuracy of the proposed method is 10–38% higher than those of the traditional methods. The recognition error of the wear debris counts decreases to 0.

## 1. Introduction

Wear of gears, bearings and other components of aero-engines is the main mechanical factor leading to engine failure and major accidents. The causes are related to material, design, environment, and working conditions. Although the causes are different, the results are that debris with wear characterization information are separated from gears or bearing parts and enter the lubrication system [1,2,3,4]. At present, the main method of wear debris detection is oil sampling analysis, which cannot meet the needs of online and real-time detection. Online detection of wear debris in Aero-engine lubrication systems can effectively detect abrupt engine wear failures and avoid major accidents. This has important theoretical significance, as well as economic and social value. The purpose of online detection of wear debris in lubricants of aero-engines is to detect abrasive particles with typical sizes of around 250 μm, and to sound an alarm when a sudden severe wear of the engine is detected. Abrasive wear characteristic information includes the material and morphology, and are related to the wear rate, type and fault source. They are generally used for off-line analysis of wear fault mechanisms and fault location diagnosis [5,6]. The number and size of wear debris are related to the severity and speed of wear, so these two characteristics are selected for online early warning detection [7].

The new generation of aero-engines uses more continuously variable transmission gearboxes and common-cavity bearings. Since common lubrication systems use multiple gears and bearings, especially in the independent transmissions, the load and working conditions are different. As a result, wear debris dislodged from different mechanical parts coexist in the lubrication system oil of the common chamber, which will be detected along with the oil flowing through the sensing detection region at the same time. The statistical rules that correspond to the distribution of wear debris sizes of a single component are no longer indicative of the total distribution.

Discerning the representativeness of a sampling of wear debris from multiple sources in new aero-engines has become an urgent technical challenge [8,9]. In recent years, researchers have studied the detection methods of lubricating oil wear debris, making use of the different physical effects with ultrasound methods, optical methods, and electrical tests. Ultrasound sensors [10] cannot distinguish bubbles in lubricating oil. Optical methods [11] are ineffective in opaque oils, and have no ability to distinguish water droplets and impurities. Many other methods, such as inductance and charge methods [12,13,14], cannot achieve multi-wear debris resolution.

Capacitive sensors can detect the change of dielectric constants between plates. Designed sensor arrays are used to measure the voltage between sensitive electrodes, and an appropriate image reconstruction algorithm is used to construct the medium distribution in the target field [15,16,17]. The dielectric constants of metallic materials differ by more than seven orders of magnitude from those of bubbles, water, oil, and other non-metallic materials [18].

In the new applications of aero-engine lubricant wear debris detection, the array capacitance method has not been applied to sparse micron-scale piecewise multi-induction-Dirac-boundary (scale→0, dielectric→∞) measurement reconstruction and feature recognition detection.

The partial differential equation is nonharmonic. It does not satisfy the linear superposition principle. The conventional linearization or regularization optimization methods, which rely on gradient or gradient norm, must pseudo-converge to the local optimal solution in the non-convex solution space. As for meta-heuristic algorithms, neural network needs a large number of training samples which is difficult to obtain from aero-engines. The Monte Carlo simulated annealing algorithm is unacceptable because of time-wasting due to the random search strategy. Genetic algorithms and group heuristic algorithms do not need gradient information. The prior knowledge, such as sensor materials and geometric parameters, is introduced through adaptive parameters. On this basis, high-level a priori knowledge, such as the topological and statistical characteristics of wear debris, can be introduced to process the inverted image topological morphology. Therefore, the hyper-heuristic algorithm can greatly improve the efficiency on the premise of guaranteeing global convergence.

The structure of this paper is as follows. Firstly, a capacitance array is proposed to detect micro-scale metal wear debris at multiple scales using Dirac-sparse and fragmented mixed media. A pole detection model of multi-induction secondary sources is established and verified by simulation. The model addresses the multiple iso-surface PDE inversion problem, where the traditional optimization method easily converges. To solve this problem, a hyper-heuristic method is proposed, combining prior knowledge of the sensor structure materials’ Low-Level Heuristics (LLH) SA-CGA inversion with the number and morphology of wear debris extracted using a High-Level Strategy (HLS) image processing algorithm. Finally, a 12-plate capacitance array sensor and detection system are designed and compared with optical microscopy off-line methods. The results show that when the size of abrasive particles is 200–900 μm and the distribution of 1–3 wear debris is different, the accuracy of the scale measurement and the recognition accuracy of the number of abrasive particles in this method are obviously improved when compared to traditional optimization methods.

## 2. Model

**Figure 1:**
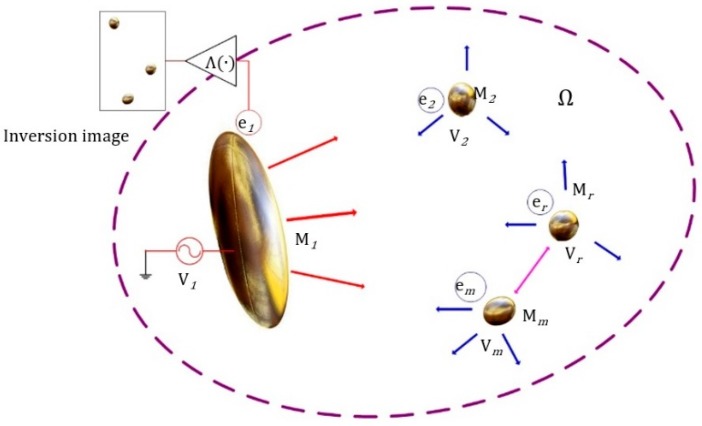
Mechanism of capacitance detection of multi-metal wear debris.

As shown in Figure 1, in region Ω, the electric potential of M_1_ is V_1_ and the free charge is e_1_. *m* − 1 metal wear debris are denoted as M_2_…M*_r_*…M*_m_*, with charges e_2_…e*_i_*…e*_m_*, and surface electric potentials V_2_…V*_r_*…V*_m_*. The oil medium between the inner plate and wear debris in the region Ω is uniformly distributed and electrically neutral. Multiple metal wear debris are affected by electric fields to form inductive secondary sources. The change of capacitance (ΔC_1_ = Δe_1_/V_1_) of the plate is detected. The location of the exciting plate M_1_ in the region Ω is changed to obtain multi-degree detection information. The image, obtained by the distribution of electrical parameters in the field, is used to quantitatively identify the wear information of the geometric characterization, where Λ(·) represents the imaging inversion operator.

In the plate capacitance array imaging system, each wear debris is uniquely determined by three independent unknown variables, the scale $a_{r}$ and abrasion locations $(\rho_{r}$, $\theta_{r})$. The number of wear debris m is also an unknown variable, with a total of 3m + 1 independent unknown variables. Array plates with different structural parameters are arranged on the oil pipeline to excite the plates with different widths R Δθ and position parameters $\dot{\theta }$~$\ddot{\theta }$. Different independent measurement data can be obtained from different angles. When the degree of freedom of the data is greater than or equal to the unknown degree of freedom of the wear debris, an appropriate inversion can be made.

**Figure 2:**
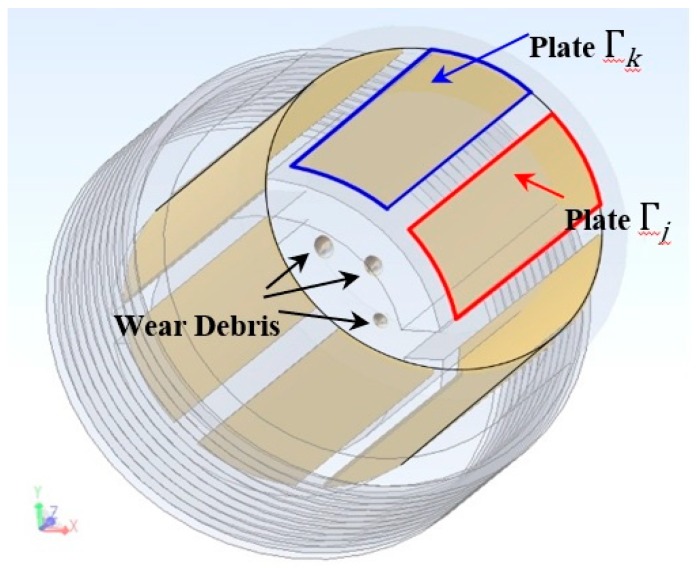
Detection of multi-wear debris capacitance array.

As shown in Figure 2, on the boundary Γ⊂∂Ω, the disconnected plates are arranged $\Gamma =\Gamma_{1}\cup \Gamma_{2},\;\ldots ,{\;\Gamma }_{j},\ldots ,\Gamma_{k},\ldots \cup \Gamma_{N}$ where *N* is the number of array plates.

Let $\Gamma_{j}=V_{p}/2$, $\Gamma_{k}=-V_{p}/2$, i.e., the excitation voltage between $\Gamma_{j}-\Gamma_{k}$ is $V_{p}$. The capacitance of the plate pair $\Gamma_{j}-\Gamma_{k}$ changes:

(1) $${\Delta C}_{j,k}=\frac{\varepsilon_{oil}}{\pi }\overset{r=m}{\underset{r=1}{\sum }}(\zeta_{r}\varsigma_{r}\xi_{r})$$

where the location factor $\zeta_{r}\;=\;{\left\{\left[F_{r}\left({\ddot{\theta }}_{j}\right)-F_{r}\left({\dot{\theta }}_{j}\right)\right]-\left[F_{r}\left({\ddot{\theta }}_{k}\right)-F_{r}\left({\dot{\theta }}_{k}\right)\right]\right\}}^{2}$, scale factor $\xi_{r}={\left(a_{r}\right)}^{2}$, and induction field factor are:

(2) $$\varsigma_{r}=1+\overset{t=\left(r-1\right)}{\underset{t=1}{\sum }}\{\frac{\left[F_{t}\left({\ddot{\theta }}_{j}\right)-F_{t}\left({\dot{\theta }}_{j}\right)\right]-\left[F_{t}\left({\ddot{\theta }}_{k}\right)-F_{t}\left({\dot{\theta }}_{k}\right)\right]}{\left[F_{r}\left({\ddot{\theta }}_{j}\right)-F_{r}\left({\dot{\theta }}_{j}\right)\right]-\left[F_{r}\left({\ddot{\theta }}_{k}\right)-F_{r}\left({\dot{\theta }}_{k}\right)\right]}\frac{{\left(a_{t}\right)}^{2}}{R_{rt}}\}$$

The array capacitance detection system of *N* cylindrical arc-shaped plates uses a voltage source $U_{p}$ excitation, and the detection interval flows through *M* wear debris simultaneously. The detection value of the array plate capacitance corresponding to changes is a N×N matrix:

(3) $$\Delta C={\left[\Delta C_{j,k}\right]}_{N\times N}$$

This is directly proportional to the dielectric constant of the background oil, and is affected by the position factor vector $\left[\zeta_{r}\right]$, the non-linear induction field effect matrix $diag[\varsigma_{r}]$ and the scale factor vector $\left[\xi_{r}\right]$. Among them, the scale factor vectors $\left[\xi_{r}\right]$ contain $a_{1},\;a_{2},\ldots ,\;a_{m}$ and *m*, which are all target parameters of wear characteristics.

The solution of this variational problem is a generalized solution, which is converted to the extremum problem under the variational method and solved using optimization theory.

The exact value $\bar{u}\;=\;K\bar{\varepsilon }$ of equation Kε=u is unknown. Given the approximate value $u_{\delta }$, it satisfies $\|\bar{u}-u_{\delta }\|<\delta$, and δ>0. The regularization operator $K_{h}:Q\to U$ in the family of continuous operators $R_{\alpha }$ satisfying the regularization conditions has deviation $\|K_{h}-\;K\|\leq h$, and h≥0. The corresponding minimization problem can be constructed.

(4) $$\min\left(\|K_{h}\varepsilon -u_{\delta }{\|}^{2}+\alpha N\left(\varepsilon \right)\right)$$

In the current regularization optimization method, α>0 is the regularization parameter and N(ε) is the regularization stability operator. The error $\left\|K_{h}\varepsilon -u_{\delta }\right\|$ is a convex function, and the regularization stabilization operator takes different functions to form different solutions. Modern optimization inversion methods adopt smoothing functions based on norms [19], i.e., regularization solutions add filtering factors on the basis of least-squares methods or filtering high frequency components to achieve regularization. For more information on the physical model of the capacitance array detection, please refer to the Supplementary Materials. 

However, there are many induced Dirac singularities in the aero-engine lubricant wear debris capacitance array. When the high-frequency component is filtered out, the relative difference of energy of the multi-Dirac function is lower than the convex functional energy, and the boundary information of the lubricant abrasive metal is lost. Subsequently, the optimization method is not suitable for the new application of multi-induced Dirac parameter reconstruction. The contrast changes sharply near the discontinuous boundary of metal abrasive particles and the solution is unstable [20].

## 3. Finite Element Analysis

As discussed in the previous section, the parameters needed for the inversion of the capacitance array detection system include the size, number and location of the wear debris. In this section, we use 3D finite-element simulation to study the influence of different wear characteristics on the test results. In the simulation model, the thickness of the tube wall is 2 mm, the diameter of the inner tube wall is 16.5 mm, the diameter of the inner core of the capacitive sensor is 9 mm, the length of the plate is 50 mm, and the number of plate arrays is 12. The typical wear debris size of sudden severe wear of aero-engine bearings ranges from 250–900 μm. The maximum velocity of wear debris is typically 200 particles per minute, which is equal to several (1–3) passing through the sensor at the same time, statistically. The relative dielectric constant of abrasive particles is set to 10^7^. The values of the simulation analysis parameters are shown in Table 1.

As the size of wear debris decreases, the position between the wear debris and the plate recedes from the center of the plate gap, the distance between the abrasive particles and the plate increases, and the detection of the response signal decreases. The relationships between the detection response and the parameters of the wear debris size, the distance between wear debris, and the plate gap are all approximately linear, and the relationship between the detection response and the distance between the wear debris and plate is approximately exponential. The model is consistent.

The sensitivity distribution of wear debris is described by the following equations:

(5) $$S{\left(a_{r}\right)}_{j,k}=2\pi {\varepsilon_{oil}}^{2}{\left(a_{r}\right)}^{2}{\left(\phi_{r}\right)}^{2}\varsigma_{r}$$

(6) $$S{\left(\theta_{r}\right)}_{j,k}=\varepsilon_{oil}\overset{r=m}{\underset{r=1}{\sum }}\left[T_{\theta_{r}}\left(\frac{\partial \phi_{r}}{\partial \theta_{r}},\frac{\partial \left(\frac{\phi_{r}}{\phi_{t}}\right)}{\partial \theta_{r}},\frac{\partial R_{rt}}{\partial \theta_{r}}\right){\left(a_{r}\right)}^{2}\right]$$

(7) $$S{\left(\rho_{r}\right)}_{j,k}=\varepsilon_{oil}\overset{r=m}{\underset{r=1}{\sum }}\left[T_{\rho_{r}}\left(\frac{\partial \phi_{r}}{\partial \rho_{r}},\frac{\partial \left(\frac{\phi_{r}}{\phi_{t}}\right)}{\partial \rho_{r}},\frac{\partial R_{rt}}{\partial \rho_{r}}\right){\left(a_{r}\right)}^{2}\right]$$

In the equation, $S{\left(a_{r}\right)}_{j,k}$ is the size sensitivity of wear debris, $S{\left(\theta_{r}\right)}_{j,k}$ and $S{\left(\rho_{r}\right)}_{j,k}$ are the sensitivity of wear debris varying with angle and position. For more detailed explanation of the sensitivity of the capacitance array, refer to the Supplementary Materials.

**Figure 3:**
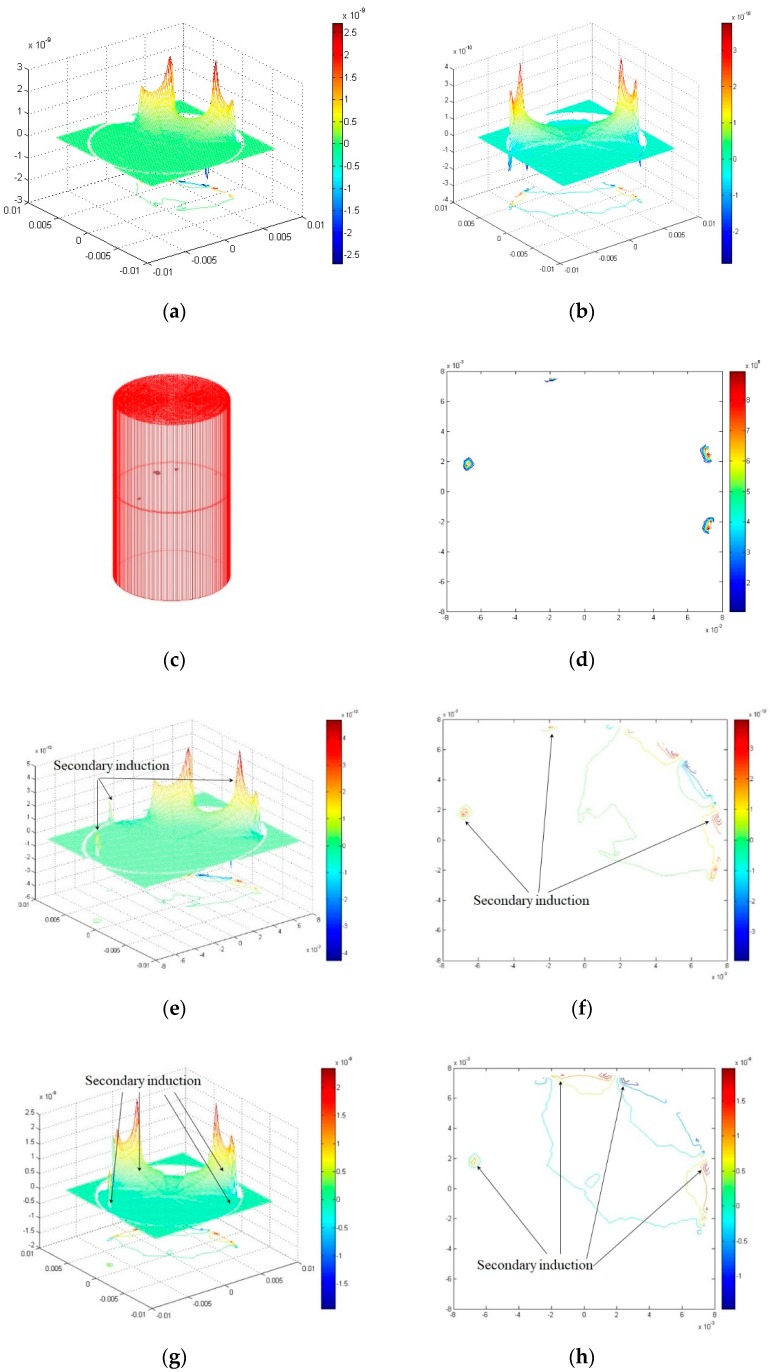
Simulation results of sensitivity field distribution. (**a**) Non-debris sensitivity fields adjacent to the plate. (**b**) Non-debris sensitivity field far from the plate. (**c**) 3D finite element models of multiple debris sources. (**d**) Abrasive induction field profile. (**e**) Multi-debris adjacent plate sensitivity field. (**f**) Multi-debris adjacent plate sensitivity field profile. (**g**) Multi-debris sensitivity field far from the plate. (**h**) Multi-debris sensitivity field profile far from the plate.

The arbitrary induced-field distribution $\varsigma_{r}$ > 1 is the minimum sensitivity of wear debris size, which can be expressed by a conformal transformation, equivalent to a uniform field single abrasive sensitivity. As shown in Figure 3, the sensitivity field distribution of the typical non-debris plate (15° of plate angle) and the non-debris plate (90° of plate angle) mirror each other. When there are many wear debris (Figure 3c), the induced field distribution of secondary source effects is shown in Figure 3b because the wear debris are metal materials. When there is more than one wear debris in the sensor detection region, the sensitivity field of capacitance arrays with different distributions of adjacent plates that are far from the plates will exhibit corresponding secondary induction changes, described in Equations (5)–(7), and shown in Figure 3e,g. This results in a distribution of the sensitivity field in the detection range of the capacitive array sensor, which is related to all the wear debris tested, as shown in Figure 3f,h.

**Figure 4:**
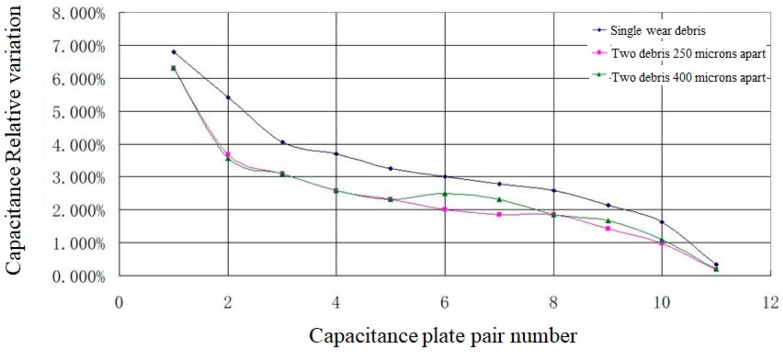
Wear debris Resolution with Different Distributions.

The simulation results of traversing typical distributions of abrasive particles show that the distance between two wear debris in the vertical direction is not distinguishable, as shown in Figure 4. The larger the size of the plate is, the larger the response signal is. The smaller the response signal is, the smaller the size of the plate is, thus, the more difficult it is to distinguish the one closest to the plate and a big debris from another small debris far from the plate.

## 4. Method

### 4.1. SA-CGA Inversion

Inversion of the capacitance array of wear debris in lubricating oils is a non-convex problem in the non-monotonic solution space of multi-induction Dirac poles. The distributions of oil in continuous and abrasive discrete phases are random, and the smoothness of the problem is unknown. The genetic algorithm (GA) optimization process does not depend on gradient information [21], and is suitable for dealing with complex non-linear problems. Cellular automata (CA) is an emergent computing method [22]. Combining the genetic algorithm of CA, it can better simulate the non-linear dynamic state of individual interactions of genetic algorithm populations, and it can also use the algorithm to introduce prior knowledge to achieve adaptive heuristic algorithms.

The calculation steps of the SA-CGA algorithm for inverse solutions of capacitance array imaging of wear debris in lubricating oil abrasive arrays are as follows:

(1) Population initialization

To a genetic algorithm cell individual $V_{i,j}\left(i,j=1,2\ldots ,n\right)$, each individual has a chromosome $x_{i,j}$, where *i*, *j* represent individual coordinates, $x_{i,j}\in \left[a,b\right]$.

(2) Coding

According to the accuracy of the calculation, the n×n mesh of variables *x* is partitioned, which belongs to a region of Ω. The minimum integer of $\left(b-a\right)\cdot 10^{n}\leq 2^{m}-1$ is expressed by *m*, then $x_{i,j}$ can be expressed with a binary string, and the dielectric constant of the corresponding parameter space is expressed by a genetic algorithm gene.

(3) Computational fitness

The gene expression of the individual $V_{i,j}$ in the cell space population is calculated, that is, the fitness eval ($V_{i,j}$) of dielectric constant value. The mathematical model of imaging detection of capacitance arrays of wear debris in lubricating oil is the change of the capacitance array calculated using a forward 3D finite element method and the coincidence of the measured value.

(4) Learning select operations

Each individual $V_{i,j}$ in the cell space evaluates the fitness of its neighbors (including itself) whose fitness is greater than or equal to its own cell (denoted as Θ) according to the difference between the neighbor form and radius in two-dimensional space.

(8) $$p_{a,b}=\frac{\mathrm{eval}\left(V_{a,b}\right)}{\sum_{a,b\in \Theta }\mathrm{eval}\left(V_{a,b}\right)}$$

where, $p_{a,b}$ is the probability that the neighbor $V_{a,b}$ of individual $V_{i,j}$ is selected (if $\mathrm{eval}\;\left(V_{a,b}\right)<\mathrm{eval}\;\left(V_{i,j}\right)$). Unlike traditional genetic algorithms, the individuals involved in the selection are limited to the individual and the set of neighbors Θ, which is the process that the central cell learns from the neighbor.

(5) Heuristic crossover

When the individual $V_{a,b}$ of the neighboring cell is selected by the individual $V_{i,j}$ of the central cell, and the crossover probability is defined as $P_{c}$, the individual $V_{i,j}$ updates the chromosome in the following way.

(9) $$x_{i,j}^{\prime }=\left(1-P_{c}\right)x_{i,j}+P_{c}x_{a,b}$$

The cross probability $P_{c}$ of the cellular genetic algorithm is different from the cross probability of traditional genetic algorithms. This can be regarded as the “confidence” or “learning tendency” of individual $V_{i,j}$. When $P_{c}$ = 1, individual $V_{i,j}$ learns aggressively from the better individuals in the neighborhood, while when $P_{c}$ = 0, individual $V_{i,j}$ is completely confident and has no confidence in its neighbors. The interest interaction in Equation (19) represents the crossover probability at a fixed value. All individuals adopt the same crossover probability, which results in the same crossover learning probability regardless of the “excellence” of their neighbors. Using this heuristic strategy, the crossover probability has a self-adaptability based on heuristic learning, which is similar to the effect of “learning intensity”.

(10) $$x_{i,j}^{\prime }=\left(1-P_{c,i,j}\right)x_{i,j}+P_{c,i,j}\left(1-P_{c,i,j}\right)x_{a,b}+{P_{c,i,j}}^{2}x^{*}$$

(11) $$P_{c,i,j}=\{\begin{array}{cc} P_{c}+\left(1-P_{c}\right)K_{1}\tanh\left\{\frac{\mathrm{eval}\left(V_{a,b}^{*}\right)-\mathrm{eval}\left(V_{i,j}^{*}\right)\cdot P_{c}}{K_{2}\left[\mathrm{eval}\left(V_{i,j}^{*}\right)-\mathrm{eval}\left(V_{a,b}^{*}\right)\right]}\right\} & ,\left(\mathrm{eval}\left(V_{i,j}^{*}\right)\neq \mathrm{eval}\left(V_{a,b}^{*}\right)\right)\\ P_{c} & ,\left(\mathrm{eval}\left(V_{i,j}^{*}\right)=\mathrm{eval}\left(V_{a,b}^{*}\right)\right) \end{array}$$

where, $K_{1}$ and $K_{2}$ are adaptive heuristic coefficients of learning intensity, which can realize the adaptability of heuristic learning intensity [23]. Without sacrificing the convergence speed, the solution quality is improved.

(6) Adaptive heuristic mutation

Here we define the mutation probability $p_{m}$. For individual $V_{i,j}$ in cell space, the random number $r_{\varepsilon }$ is selected from [0,1]. If $r_{\varepsilon }<p_{m}$, the chromosome of the individual is updated as follows.

(12) $$x_{i,j}^{\prime \prime }=x_{i,j}^{\prime }+\delta_{\varepsilon }\phi$$

Among them, $\delta_{\varepsilon }$ is constant, ϕ~N(0,1).

Traditional genetic algorithms define the mutation probability $p_{m}$ as a constant, and its selection often depends on empirical values. The mutation probability is large, and the global search ability is strong, but the search speed is slow. The probability of mutation is small, the convergence rate is fast, the diversity of the population is poor, and meaningless local solutions are obtained. The selection of actual constants is a compromise of the empirical value at the expense of adaptability. The mutation probability is adjusted adaptively.

(13) $$p_{m}^{\prime \prime }=1-{\left(1-p_{m}^{\prime }\right)}^{{\left(T_{R}\right)}^{2}}$$

where, $p_{m}^{\prime }$ is the mutation probability of the previous population generation, $p_{m}^{\prime \prime }$ is the new mutation probability when the local population undergoes mutation operations, and $T_{R}$ is the population of the superposition of the optimal elite under the strategy of retaining the elite, that is, the local optimal continuous reproduction algebra.

The initial value of mutation probability is a very small original mutation probability $p_{m}$. When the optimal elite passes through the iteration, new locally optimum points with higher adaptability are found, and the mutation rate is restored to the initial value $p_{m}$. Through adaptive variation of the mutation probability, the mutation rate is related to the number that fall into local optima. The longer the time of iteration, the greater the mutation probability, thus it accelerates exponentially. The convergence of the algorithm is good in most cases, and the search for all optimum points can be accelerated when the iteration falls into a local optimum.

(7) Determination of whether criteria for evaluating biological groups are met; if not, return to step 3.

Generally, the fitness of selected populations is larger than a certain value, and the increasing rate of the population fitness does not increase with the number of evolutions or reach a certain number of evolutions. Rudolph proves that the elite-preserving genetic algorithms converge to the global optimal solution with probability 1, based on Markov chain theory. The strategy of retaining the elite refers to the strategy of retaining the best individuals in the population to the next generation in the selection operator. This strategy does not participate in crossover and mutation operations, so as to prevent the excellent individuals in each generation from losing due to crossover and mutation operations, thus falling into local extrema. This strategy is introduced into the cellular genetic algorithm. In the crossover operation, the individual cell not only learns from the excellent neighbors, but is also influenced by the contemporary global optimum. A new crossover operation model is proposed.

(14) $$x_{i,j}^{\prime }=\left(1-P_{c}\right)x_{i,j}+P_{c}\left(1-P_{c}\right)x_{a,b}+{P_{c}}^{2}x^{*}$$

where $x^{*}$ indicates that the maximum fitness of the contemporary population corresponds to the chromosome of the individual. Cellular individuals are affected not only by the excellent neighbors around them, but also by the best individuals in the contemporary population.

**Figure 5:**
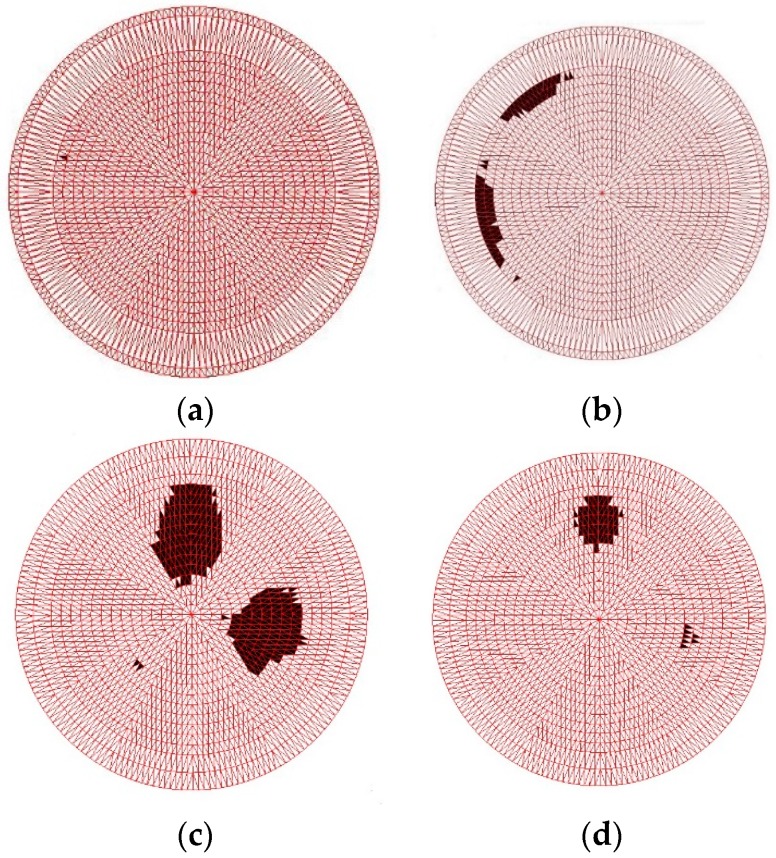
SA-CGA inversion results: (**a**) Single debris (250 μm). (**b**) Two debris. (**c**) Three debris (2 big and 1 small). (**d**) Three debris (1 big and 2 small).

The fitness of each individual in the population is calculated using 3D-FEM. To ensure the accuracy of the inversion solution of the capacitance array of lubricating wear debris, a non-linear calculation model is adopted, and the calculation time cost is the highest. Therefore, before evaluating the fitness of biological groups, the heuristic algorithm [24,25] is implemented by introducing the prior knowledge of tribological statistics (such as the number and size range of wear debris) to improve the results of obvious non-conformity with a small computational cost. Using the SA-CGA algorithm, the inversion results of measured signals are shown in Figure 5.

Figure 5b shows two 900 μm wear debris and one 200 μm wear debris separated by a plate. The inversion calculation has artifacts but can distinguish them. For two particles with similar size at different times on a plate (for instance, in Figure 5d where two 200 μm particles and one 900 μm particle are separated by one plate), only two particles are displayed after inversion. This is because the size of the particle is large, although the other is far away from the three particles, which affects the threshold decision of the debris judgment strategy. In the next section, a High-Level Strategy (HLS) heuristic algorithm will be used to optimize the detection effects by utilizing the prior statistical knowledge of local morphology of wear debris.

### 4.2. Morphological Hyper-Heuristic

**Figure 6:**
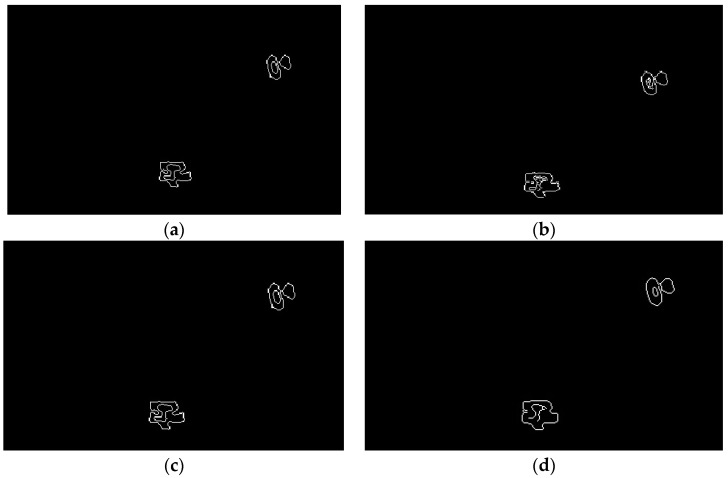
Different operators on wear debris edge hyper-heuristic detection (**a**) Sobel operator. (**b**) Prewitt operator. (**c**) LOG operator. (**d**) Canny operator.

Combining the prior knowledge of piecewise constants of wear debris distributions and effective pixel-sparse statistical features, while using the local or global differential or gray distribution information, the features of the boundary between the debris object and the oil background can be identified. The key point is to identify the boundary in the case of low signal-to-noise ratios and avoid the artifacts of the multilateral boundary as far as possible. The heuristic intelligent-image edge-detection algorithm, using the local second derivative information of the image, combined with intelligent filtering, has better adaptability for gray image processing with noise, such as in the case of the Laplacian-Gauss (LOG) operator. The effect of different edge extraction operators is verified by the 3-debris image of Figure 5d, which is the most difficult to distinguish, as shown in Figure 6.

The Sobel and Prewitt operators only segmented two non-connected regions of the actual distribution of the gray image where the number of wear debris was 3. The number of image segmentations is obviously not consistent with the number of wear debris. In the edge-recognition and segmentation algorithms for the three non-connected regions, the LOG operator uses a two-pixel boundary. The Canny algorithm uses a double-threshold operator to overcome this problem. After edge recognition and segmentation with a Canny operator, the number of oil and wear debris detection and size target features can be extracted. The number of wear debris is the number of non-connected segmentation areas in image morphology, and the size of abrasive particles is the fitted line length of image using a mathematical morphology skeleton.

For the image with a recognized boundary, the wear debris region is obtained with a filling operation. Let the set ***A*** of region boundaries be complemented by $A^{c}$. The region can be filled by expanding, complementing, and exchanging the structure element ***B*** (morphological mapping of abrasive particle inversion image). Firstly, a point in the boundary is assigned a value of 1, according to the iteration formula.

(15) $$X_{k}=\left(X_{k-1}⊕B\right)\cap A^{c}k=1,2,3$$

We fill in these values, where the last iteration result of the image set is $X_{k-1}$, and the solution result is $X_{k}$. When $X_{k-1}=X_{k}$, the iteration stops; ⊕ is an expansion mapping operation, and ∩ is an intersection mapping operation. According to the minimum size of wear debris judged by the target of lubricating oil, the size of the wear particles is 250 μm. The corresponding image is a 10×10 pixel spatial area. According to the prior knowledge of the shape of the wear particles, the structural element is defined as a circle.

According to the typical hyper-heuristic knowledge of aero-engine fatigue wear debris, the shape boundary of wear debris satisfies Lipschitz smoothness, so the image of wear debris is de-sharpened. Using a closed mapping of the set algebra, the image expands and corrodes again. On the premise of maintaining the area and shape characteristics of the wear debris image, the wear debris boundary is de-sharpened and defined as:

(16) D=A·B=(A⊕B)⊖B

where ⊕ is the expansion operator of image mathematical morphology and ⊖ is the image mathematical morphology corrosion operator.

The actual size of the wear debris corresponds to the characteristics of the length of the central axis in the non-connected areas of the image:

(17) $$R_{M}=L\left[S\left(D\right)\right]$$

where $R_{M}$ is the actual size of the abrasive particles, L is the length feature, and S(D) is the skeleton of the non-connected area of the image after deburring by the operations of Equation (26).

(18) $$S\left(D\right)=\cup_{k=0}^{K}\;S_{k}\left(D\right)=\left(D⊖kB\right)-\left[\left(D⊖kB\right)⋄B\right]$$

Here, $S_{k}\left(D\right)$ is the skeleton subset, **B** is the structural element, (D⊖kB) represents the continuous *k*-times **B**-to-**D** corrosion operation, and **K** is the last iteration number before **D** is corroded into an empty set.

According to Equation (28), the main factor affecting the conversion relationship between the actual size of wear debris and image features is the selection of structural element **B**. **B** is too small to cause connectivity damage, but **B** is too large to easily lose skeleton branch details of the skeleton. The details of skeleton branches do not affect the calculation of skeleton length, but the failure of skeleton connectivity affects the calculation of skeleton length. Therefore, the **B** selection strategy should be the maximum structural element with the resolution allowed. According to the minimum target resolution pixel of 250 μm for abrasive particles, 10×10 structural elements are selected.

After wear debris edge recognition, it is necessary to fill in the internal pixels according to the outermost layer recognized as the edge of the wear debris. This kind of wear debris recognition filling can use mathematical morphology algorithms and basic operations combining processing with set theory to extract the corresponding morphological structure characteristics of wear debris. For images with recognized boundaries, the debris region is obtained by a filling operation. The number of wear debris can be determined by checking the connectivity between each pixel and adjacent pixels. The number of pixels in the image itself is small, and the number of abrasive particles can be measured using the pixel marking algorithm. When labeling in detail, the binary image after edge recognition and boundary determination is scanned from left to right and from top to bottom.

**Figure 7:**
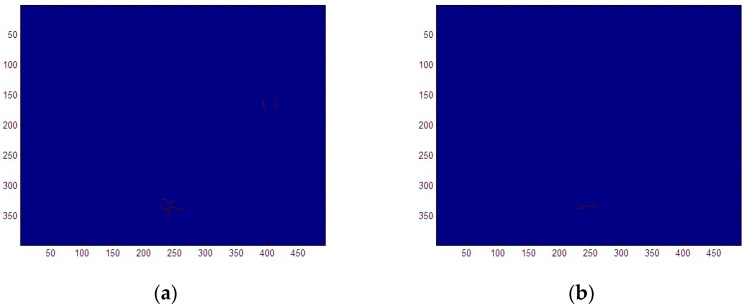
Abrasive hyper-heuristic skeleton extraction. (**a**) Direct skeleton extraction of wear debris. (**b**) Mathematical morphology hyper-heuristic skeleton extraction.

The image processing recognition and length measurement are carried out using the central axis of different wear debris in the image, and the debris size measurement is achieved using morphological skeleton extraction algorithms. Due to the edge recognition and image filling of wear debris, the shapes of debris edges are irregular, and there are protrusions and concave angles on the edges of wear debris. Figure 7a demonstrates how the mathematical morphological skeleton is obtained, where the edge is very irregular, resulting in skeleton bifurcation, which cannot effectively measure the size of wear debris. The typical shape boundary of aero-engine fatigue wear debris is used to satisfy the Lipschitz-smooth a priori hyper-heuristic criterion, and the image of wear debris is de-sharpened. Set algebraic closed mapping is used to corrode the image after expansion, and the wear debris is de-sharpened, while the area and shape features of the debris image are preserved. Through mathematical morphological closure of the debris image, the debris edge of the original irregular edge is smooth enough, considering the premise that the shape of the debris area is basically unchanged, and then a satisfactory single debris skeleton line can be obtained by calculating the skeleton of the wear debris image, as shown in Figure 7b. For the extracted debris skeleton, diagonal fitting is used to measure the wear debris size.

### 4.3. Experimental Set-Up

**Figure 8:**
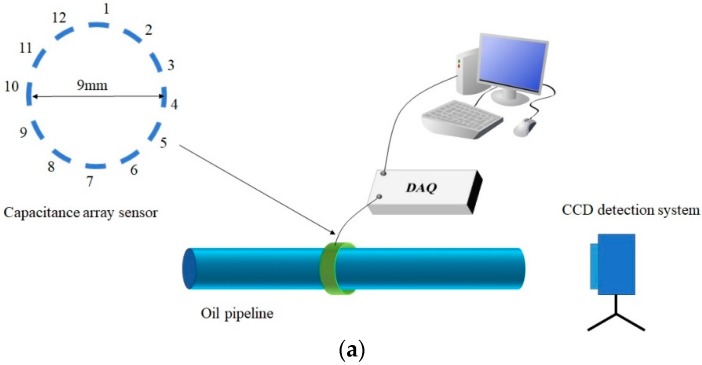

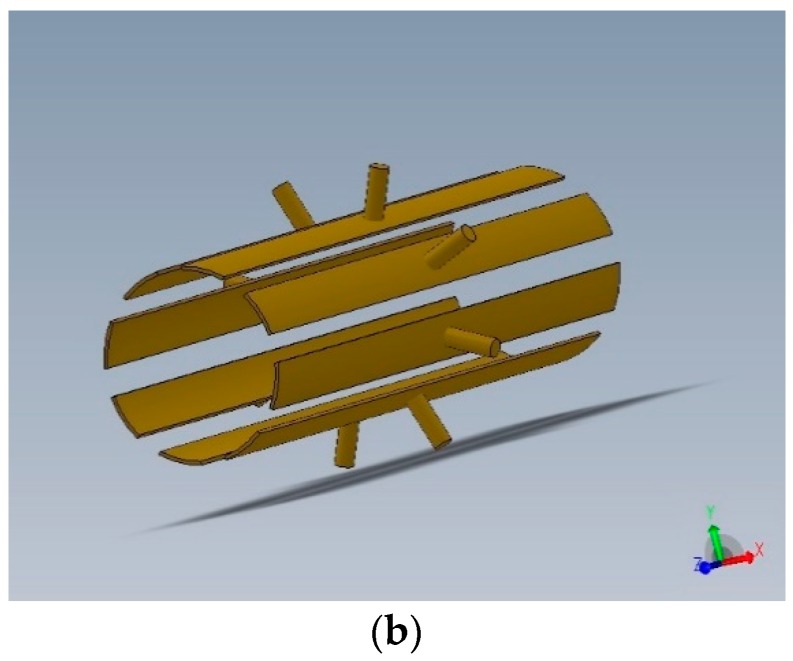
Experimental set-up. (**a**) Detection system in the experiment. (**b**) 3-D model of capacitance array sensor.

The detection system consists of hardware and software. The hardware consists of sensors on an oil pipeline, data acquisition (DAQ) circuits, cables, connectors and interface circuit boards, as shown in Figure 8a. A CCD detection system is used for comparison.

The sensor adopts 12 annular channel capacitance arrays. The diameter of a typical aero-engine oil pipeline is 8 mm. The outer diameter of the capacitance sensor is *R*. To ensure that the minimum cross-sectional area of differential pressure fluid flow is 70%, the allowable area is 150 mm^2^. Based on the area, *R* = 8.24 mm and the diameter of the capacitance sensor is 9 mm. The theoretical value of the capacitance between adjacent plates is about 2 pF. The interval between the plates is 2.3 mm. The 3-D model of the capacitance array sensor is shown in Figure 8b. The diameter is 9 mm and the length is 50 mm. When the size of the wear debris is 250 μm, the detection distance of the plate with the corresponding resolution $\Delta C_{min}$ = 0.061 fF is 5 mm.

The data acquisition (DAQ) adopts digital switched capacity technology. It is realized by ADI AD7746 chip circuit module EVAL-AD7745/46EBZ. The highest resolution is 4 aF (21 bits), and the calibration accuracy is 4 fF. The capacitance measurement range is +4 pF. To minimize the background stray capacitance of cables, joints and interface circuit boards, tetrafluoroethylene (PTFE) materials are used for sensor interface boards and joints and cables. The detection software is based on LabVIEW.

The simulated wear debris are made of standard metal particles from the CONSTANT Company, USA. The sizes of the debris are calibrated by electron microscopy and molecular sieving after calibration. Large wear debris (≥400 μm) are purchased as standard spherical abrasives. The background scale is set by a microscopic cross-shaped eyepiece micrometer (model CAT907). Small-size wear debris (<400 μm) measure the longest side according to the calibrated microscope image and determine the length-to-diameter ratio. The experimental parameters are shown in Table 2.

A CCD image (with model MV1300UC-1 of Microvision Co., Ltd., San Diego, CA, USA) of an electronic magnifier is used for comparison with the image of the capacitance array sensor. The CCD detection system is equipped with a 0.7–4.5 industrial variable lens, an 8-million-pixel CCD, and supporting software. The wear debris are calibrated with a standard micrometer. The rigid PU rod was fixed with a transparent liquid adhesive.

### 4.4. Results and Discussions

**Figure 9:**
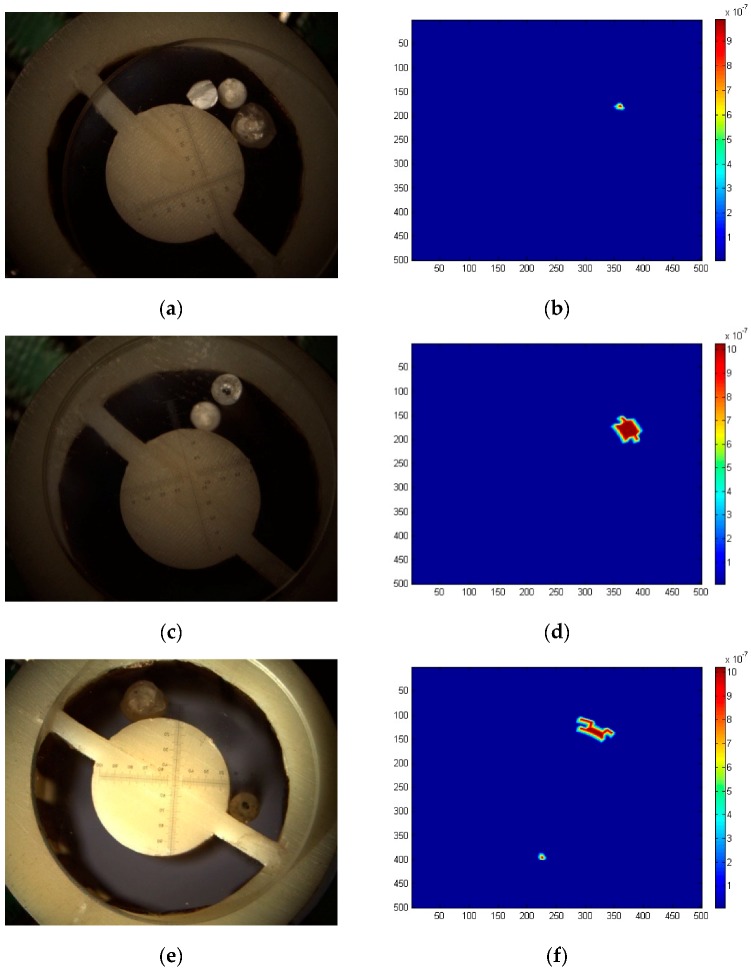

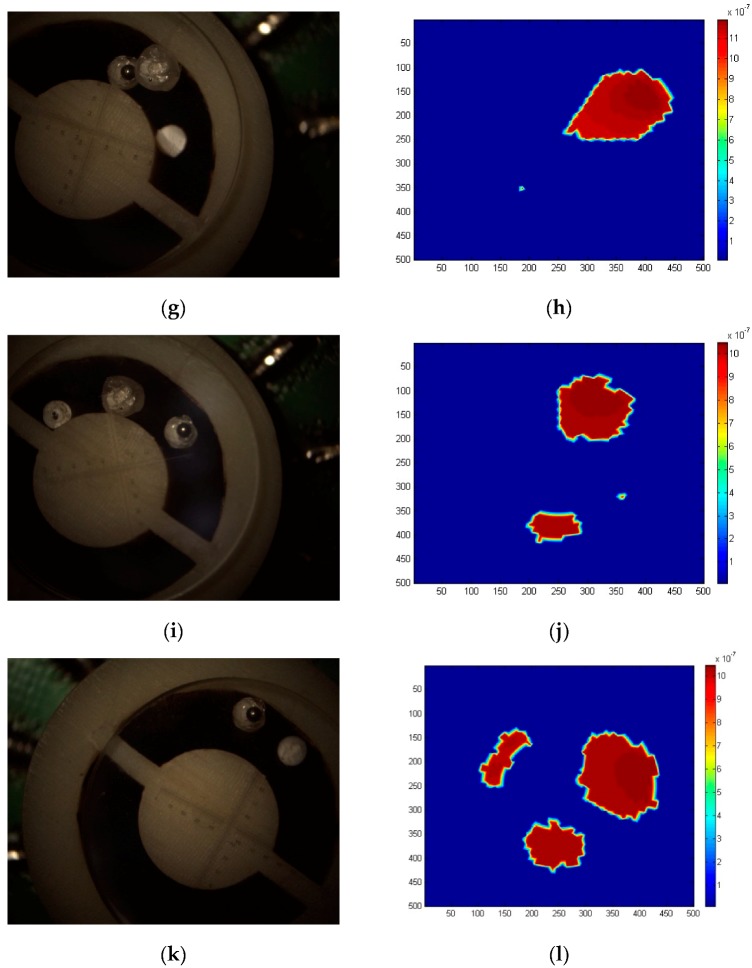
Debris imaging of CCD and capacitance array inversion methods. (**a**) Single 200 μm debris CCD image. (**c**) Single 500 μm debris CCD image. (**e**) Two small debris (separated from each other) CCD images. (**g**) Two adjacent debris (1 big and 1 small) CCD image. (**i**) Three debris (separated from each other) CCD image. (**k**) Three adjacent debris CCD image. (**b**,**d**,**f**,**h**,**j**,**l**) Corresponding capacitance array inversion.

By changing the size of wear debris that have been calibrated over 200, 900 and 500 μm, the experiments are carried out at different positions of the sensor, and the CCD image and capacitance array inversion image are measured simultaneously. The images and feature recognition outputs are compared. The image comparison results of wear debris are shown in Figure 9. In the sensor, the dielectric constant of the sensor and the plastic rod are close to that of the oil without wear debris, so the capacitance array inversion results in fluctuations lower than 2.

The results of single wear debris detection are as follows. In Figure 9a–d, the CCD image is calibrated with a stand micrometer, and the wear debris size of corresponding inversion image is monotonous. Due to the iterative synthesis calculation of array inversion, the debris edge is not smooth, and the image morphology processing does not affect the wear debris size detection. Figure 9e–h shows that when two wear debris are present at the same time, the inversion effect is obviously affected by the secondary induction field effect. The size of small wear debris near each other will increase due to the induction field, as shown in Figure 9h. The secondary induction field will distort the shape of the inverted debris, resulting in low recognition accuracy of the size of wear debris. The shape distortion effect of wear debris in the secondary induction field exists in the detection and inversion of multi-wear debris, as shown in Figure 9i–l. The array inversion calculation does not consider the distance information between wear debris. Therefore, the number and size information of wear debris in the inversion results are better, and the corresponding accuracy of the distance and relative position relationship between single wear debris and the CCD image is low. For one, two, and three pieces of debris, the error of the size inversion is lower than 9%, 15% and 17%, respectively, and for the number of abrasives, the error of the size inversion is less than 18%.

As seen in Table 3, compared with gradient or convexity optimization algorithms, the minimum relative capacitance residual of LBP or TSVD algorithm is about 1.12%. The minimum relative capacitance residual of the convexity regularization LANDWEB algorithm is about 2.01%. Continuous iteration of the LANDWEB algorithm will lead to the increase of the relative capacitance residual. SA-CGA and LFLCS hyper-heuristic algorithms are useful for improving the detection accuracy, especially when the recognition error of the number of multi-wear debris is reduced by half.

Linear gradient methods can only obtain one pole point; other secondary induction pole point information is all missing. Furthermore, when linear gradient methods are used to detect multiple pieces of wear debris, their being mistaken for single particles leads to a serious reduction in accuracy. The nonlinear gradient method falls into local solutions and produces a large number of artifacts satisfying pseudo-convergence in the sense of the least squares norm. There is little difference between heuristic algorithms and non-heuristic algorithms in terms of size accuracy for single abrasive particle detection. Simple heuristic algorithms have insufficient prior knowledge, and obvious superfluous artifacts appear in the calculation of the sensor inner core and connecting rib. The HLS knowledge of image morphology related to debris and sensors introduced in the hyper-heuristic algorithm greatly improves of number error.

The SA-CGA+LFLCS cyclic iteration hyper-heuristic strategy introduces a genetic algorithm for excellent population DNA and feeds it back into the inversion algorithm. This is better than the strategy of SA-CGA image inversion combined once, using SA-CGA+LFLCS as hyper-heuristic knowledge. Cyclic iterative processes are equivalent to the eugenic and eugenic reproductive hyper-heuristic strategies. The test results show that the accuracy of size recognition was further improved by 5%. The iterative hyper-heuristic algorithm significantly improves size accuracy. The calculation times of each algorithm are compared, as shown in Table 3. The algorithm is implemented by the software of MATLAB version 7.0.0.19920 (R14). The total code file is 49.5 MB. The running platform is a Pentium dual-core CUP E5800@3.20GHz computer, with 1.96 GB memory.

The LBP algorithm mainly calculates matrix multiplication. The TSVD algorithm mainly calculates matrix singular value decomposition. The linear gradient algorithm is a non-iterative solution, so the calculation time is short. The main computational tasks of Land-Weber are numerical integration and generalized inverse matrix. Pre-iteration of the generalized inverse matrix by off-line calculation can save computational time, and the computational time of the non-linear gradient algorithm based on this strategy is about 250 milliseconds. The calculation time of the meta-heuristic algorithm increases greatly, which is mainly because the fitness value of each iteration needs to be calculated, and the search space is greatly enlarged. In the hyper-heuristic algorithm, ONE HLS and Iteration HLS strategies have little difference in computing time. Although they increase the computational complexity of image morphological processing, the overall computing time is slightly shorter than the meta-heuristic algorithm because of the introduction of a large amount of high-level heuristic a priori knowledge, which greatly reduces the search space.

To ensure the resolution of micro-scale wear debris, the total number of three-dimensional finite element elements used in the calculation model of all algorithm is 9216. According to the characteristics of the inversion problem, the sparse matrix processing method is adopted. The peak memory usage by the algorithm is no more than 1 GB. The calculation time of each algorithm is independent of the number and size of the wear debris. 

The 32-cell hyper-heuristic algorithm was run in parallel with EPM570T100C5N (Altera) CPU (8 MHz main frequency). The computing time was about 340 milliseconds, meeting the requirement for on-line detection.

## 5. Conclusions

Based on the requirement of online detection of wear debris in aero-engine lubricating oil and micron-scale multi-wear debris, a hyper-heuristic array capacitance method for detecting aero-engine lubricating oil wear debris was proposed. We established a mathematical model using capacitance array detection for lubricating wear debris in multi-target micron-scale and sparse mixed media with constant fragmentation. A 12-pole circular channel arc capacitance array sensor was designed. A detection system and metal wear debris simulation experimental system were built. The characteristics of wear debris in the detection system were studied using finite-element simulation. The influence of characteristics on plate capacitance were considered, including the resolution of single and multiple targets. The feasibility of multi-wear debris detection using a hyper-heuristic capacitance array method was verified. The results were compared to those from the CCD imaging system. For different scales, different amounts of lubricating wear debris could be accurately detected, and the effect of the cyclic iteration strategy hyper-heuristic algorithm was demonstrated. In this paper, a hyper-heuristic capacitance array imaging method was proposed to achieve online and continuous full-flow detection of abrasive wear characteristics. We have demonstrated a technique for detecting multiple wear debris passing through at the same time to avoid false alarms. These findings have important theoretical significance and engineering application value.

## Figures and Tables

**Table 1:** Value of influence variables for simulation analysis.

Experimental Value Influence Factor	Minimum	Intermediate Values			Maximum
Wear debris size (μm)	250	300	500	700	900
Distance (mm) between debris and plate	0.5	0.5	1	1.5	2
Position of debris and Polar Plate (μm)	0	250	500	750	1000
Number of debris	1	2			3
Distance (mm)between debris	0.5	2	4	6	8

**Table 2:** Influence factors and experimental values of experiments.

Values of Experiments Influence Factors	Minimum	Intermediate Values			Maximum
Debris size (μm)	250	400	500	700	900
Debris material	Al	M50 Alloy			Cu
Morphology (Aspect ratio)	1:1	1:3			1:8
Distance (mm) between debris and plate	0.5	0.5	1	1	2
Position(μm) of debris and Polar Plate	0	250	5500	750	1000
Number of debris	1	2			3
Distance (mm)between debris	0.5	2	4	6	8

**Table 3:** Comparisons of algorithms.

Inversion Methods			Debris Distribution					
			1 Debris		2 Debris		3 Debris	
			200 μm	900 μm	200 + 900 μm		200 + 900 + 500 μm	
			Size Accuracy		Number Error	Size Accuracy	Number Error	Size Accuracy
			Calculation Time		Calculation Time		Calculation Time	
Optimization methods	linear gradient	LBP	62%	68%	−1	42%	−2	29%
			150.5 mS		150.7 mS		153.1 mS	
		TSVD	64%	71%	−1	48%	−2	32%
			213.7 mS		240.6 mS		225.3 mS	
	nonlinear gradients	Land- weber	82%	87%	6	58%	6	52%
			883.5 mS		882.7 mS		891.0 mS	
Heuristic methods	metaheuristic	SA-CGA LLH	92%	91%	3	79%	3	77%
			1001.1 mS		1009.4 mS		991.4 mS	
	hyper-heuristic	Once HLS	93%	90%	0	81%	0	85%
			761.1 mS		763.8 mS		770.6 mS	
		Iteration HLS	93%	92%	0	85%	0	90%
			771.4 mS		780.2 mS		772.9 mS	

## Supplementary Materials

The following files are available online at http://www.mdpi.com/1424-8220/19/3/515/s1.
