# Supplementary material for: Hyper-Heuristic Capacitance Array Method for Multi-Metal Wear Debris Detection
Source: Sensors (Basel). 2019 Jan 26;19(3):515. doi: 10.3390/s19030515 (PMC6387040; doi:10.3390/s19030515)
Supplement: Supplementary file 1 [file sensors-19-00515-s001.pdf]

Supplementary Materials

1. Physical model of Multi-wear debris Capacitance Array

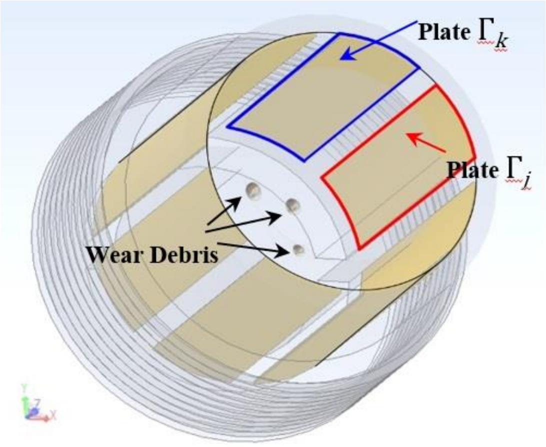

Figure S1. Detection of Multi-wear debris Capacitance Array.

In the plate capacitance array imaging system, each wear debris is uniquely determined by three independent unknown variables, the scale a_r and abrasion locations (ρ_r,θ_r). The number of wear debris m is also an unknown variable, with a total of 3m+1 independent unknown variable. Array plates with different structural parameters are arranged on the oil pipeline to excite the plates with different widths $\mathrm{R}\Delta\theta$ and position parameters $\dot{\theta}$~$\ddot{\theta}$. Different independent measurement data from different angles can be obtained. When the degree of freedom of the data is greater than or equal to the unknown degree of freedom of the wear debris, an appropriate inversion can be made.

As shown in Figure 1, on the boundary $\Gamma \subset \partial\Omega$, the disconnected plates are arranged $\Gamma = \Gamma_1 \cup \Gamma_2 \ldots \Gamma_j \ldots \Gamma_k \ldots \cup \Gamma_N$ where N is the number of array plates, the plates are uniform and the width is equal. The position of plate Γ_j and plate Γ_k are:

$$\begin{aligned} \Gamma_j: \quad & \ddot{\theta}_j = \frac{2\pi j}{N} + \frac{f}{\mathrm{R}} \quad \dot{\theta}_j = \frac{2\pi(j+1)}{N} \\ \Gamma_k: \quad & \ddot{\theta}_k = \frac{2\pi k}{N} + \frac{f}{\mathrm{R}} \quad \dot{\theta}_k = \frac{2\pi(k+1)}{N} \end{aligned} \tag{1}$$

Let $\Gamma_j = \mathrm{V}_p/2,\ \Gamma_k = -\mathrm{V}_p/2$, i.e. the plate excites between $\Gamma_j - \Gamma_k$. The distribution of the excitation of the primary field can be obtained as follows:

$$\tilde{\Phi}_{j,k}(\rho_r,\theta_r) = \frac{V_p}{\pi}\{[\mathrm{F}_r(\ddot{\theta}_j) - \mathrm{F}_r(\dot{\theta}_j)] - [\mathrm{F}_r(\ddot{\theta}_k) - \mathrm{F}_r(\dot{\theta}_k)]\} \tag{2}$$

where $\mathrm{F}_r(\theta)$ is a partial differential field equation for the electric field in a cylindrical coordinate system:

$$\mathrm{F}_r(\theta) = \mathrm{arctg}\frac{(\mathrm{R}^2-{\rho_r}^2)\sin(\theta-\theta_r)}{2\mathrm{R}\rho_r+(\mathrm{R}^2+{\rho_r}^2)\cos(\theta-\theta_r)} \tag{3}$$

The capacitance of the plate pair $\Gamma_j - \Gamma_k$ changes:

$$\Delta\mathrm{C}_{j,k} = \frac{\varepsilon_{oil}}{\pi}\sum_{r=1}^{r=m}(\zeta_r\varsigma_r\xi_r) \tag{4}$$

where the location factor $\zeta_r = \{[\mathrm{F}_r(\ddot{\theta}_j) - \mathrm{F}_r(\dot{\theta}_j)] - [\mathrm{F}_r(\ddot{\theta}_k) - \mathrm{F}_r(\dot{\theta}_k)]\}^2$, scale factor $\xi_r = (a_r)^2$, and induction field factor are:

$$\varsigma_r = 1 + \sum_{t=1}^{t=(r-1)}\{\frac{[\mathrm{F}_t(\ddot{\theta}_j)-\mathrm{F}_t(\dot{\theta}_j)]-[\mathrm{F}_t(\ddot{\theta}_k)-\mathrm{F}_t(\dot{\theta}_k)]}{[\mathrm{F}_r(\ddot{\theta}_j)-\mathrm{F}_r(\dot{\theta}_j)]-[\mathrm{F}_r(\ddot{\theta}_k)-\mathrm{F}_r(\dot{\theta}_k)]}\frac{(a_t)^2}{R_{rt}}\} \tag{5}$$

When the voltage of the k plate is U_k, the change of charge on the plate $\Delta Q_j, (j = 1,2,\ldots,N)$ can be represented in matrix form:

$$\begin{bmatrix}\Delta Q_1\\ \Delta Q_2\\ \vdots\\ \Delta Q_j\\ \vdots\\ \Delta Q_N\end{bmatrix}=\begin{bmatrix}\Delta C_{1,1} & -\Delta C_{1,2} & & -\Delta C_{1,k} & \cdots -\Delta C_{1,j}\cdots & -\Delta C_{1,N}\\ -\Delta C_{2,1} & \Delta C_{2,2} & & & \ddots & -\Delta C_{2,N}\\ & & & & \vdots & \vdots\\ & \cdots & & \Delta C_{j,k} & \cdots\ \Delta C_{j,j}\ \cdots & -\Delta C_{j,N}\\ & & & & \vdots & \vdots\\ -\Delta C_{1,k} & -\Delta C_{2,k} & \cdots & -\Delta C_{N,k} & -\Delta C_{N,j}\cdots & \Delta C_{N,N}\end{bmatrix}\begin{bmatrix}U_1\\ U_{p2}\\ \vdots\\ U_p\\ \vdots\\ U_N\end{bmatrix} \quad (6)$$

The array capacitance detection system of N cylindrical arc-shaped plates uses a voltage source U_p excitation, and the detection interval flows through M wear debris simultaneously. The detection value of the array plate capacitance corresponding to changes is a $N \times N$ matrix :

$$\boldsymbol{\Delta C} = \left[\Delta C_{j,k}\right]_{N\times N} \quad (7)$$

By Equation 5, the independent measurement values of the capacitance change $\Delta C_{j,k}$ are expressed in matrix form:

$$\Delta C_{j,k} = \frac{\varepsilon_{oil}}{\pi}\begin{bmatrix}\zeta_{1\,j,k}\\ \zeta_{2\,j,k}\\ \vdots\\ \zeta_{m\,j,k}\end{bmatrix}^T_{1\times m}\begin{bmatrix}\varsigma_{1\,j,k} & & & \\ & \varsigma_{2\,j,k} & & \\ & & \ddots & \\ & & & \varsigma_{m\,j,k}\end{bmatrix}_{m\times m}\begin{bmatrix}\xi_1\\ \xi_2\\ \vdots\\ \xi_m\end{bmatrix}_{m\times 1} \quad (8)$$

This is directly proportional to the dielectric constant of the background oil, and is affected by the position factor vector $[\zeta_r]$, the non-linear induction field effect matrix $diag[\varsigma_r]$ and the scale factor vector $[\xi_r]$. Among them, the scale factor vectors $[\xi_r]$ contain a_1, a_2, $\dots a_m$ and m, which are all wear characteristics target parameters.

When the inverse operator is continuous, a small change of u corresponds to a small change of solution ε. Under this condition, inversion using Dirichlet-to-Neumann mapping can ensure that the inversion solution is unique. To avoid an ill-posed underdetermined problem, the current method is to replace the original equation with an approximate equation containing a small parameter using a regularization technique, so that the changed equation can be solved stably, and the solution of the original equation is expected to be replaced approximately when a is very small.

In other words, there is a multi-valued operator R that maps the element u in normalized space U to a sub-column Q in normalized space Q. Approximate sub-columns of Q are defined by $v(Q)$=$Q^{\#}$ and the distance d between the two sub-columns Q and Q is defined as:

$$\sup_x \inf_v \|x - v\|_X + \sup_v \inf_x \|x - v\|_X \quad (9)$$

In the first term, the supremum is relative to $v \in Q^{\#}$, the infimum is relative to $x \in$ Q, and the second term exchanges x and v. If $\|u^{'} - u\|_{TT}$ approaches zero, $d(R\varepsilon^{'}, R\varepsilon)$ approaches zero, and the multi-valued operator R is continuous. The adjacent U mapping U_M to a family of continuous operatorsR_α of $v(Q)$ is called the regularization operator of the equation $\mathbf{K}\varepsilon = u$ if the following conditions are satisfied:

$$\lim_{\alpha\to 0} \mathrm{R}_\alpha \mathrm{K}\,\varepsilon = \varepsilon \forall UM \quad (10)$$

where the positive coefficient α is referred to a regularization parameter.

In the simply connected bounded domain $\Omega \subset \mathrm{R}^n$, $n \geq 2$, the equations satisfying the corresponding excitation and non-local boundary conditions of metal wear debris are solved. According to the variational principle, if $u = u(x)$ is a classical solution, u is continuous on $\overline{\Omega}$ and piecewise continuously differentiable, and quadratic continuously differentiable Ω, then u must be in the class of functions:

$$\tilde{V} = \begin{cases} v \text{ (Continuous and differentiable)} \\ v|_{\Gamma_1} = \mathrm{U_{in}}, v|_{\partial C_k(k=1,\dots\dots p)} = U_k \text{(Constant)} \end{cases} \quad (11)$$

We obtain these functionals:

$$\mathcal{J}(v) = \int_{\Omega} \frac{1}{2}|\mathrm{grad}v|^2 \mathrm{d}x - A_0 v|_{\partial C_k(k=1,\dots p)} \quad (12)$$

The minimum value is referred to as:

$$\mathcal{J}(u) = \inf_{v \in \bar{V}} \mathcal{J}(v) \quad (13)$$

The solution of this variational problem is a generalized solution, which is converted to the extremum problem under the variational method and solved using optimization theory.

The exact value $\bar{u}$=$\mathbf{K}\bar{\varepsilon}$ of equation $\mathbf{K}\varepsilon = u$ is unknown. Given the approximate value u_{δ}, it satisfies $\|\bar{u} - u_{\delta}\| < \delta$, and $\delta > 0$. The regularization operator $\mathbf{K}_h: Q \to U$ in the family of continuous operators R_{α} satisfying the regularization conditions has deviation $\|\mathbf{K}_h - \mathbf{K}\| \leq h$, and $h \geq 0$. The corresponding minimization problem can be constructed.

$$\min(\|\mathbf{K}_h\varepsilon - u_{\delta}\|^2 + \alpha N(\varepsilon)) \quad (14)$$

In the current regularization optimization method, $\alpha > 0$ is the regularization parameter and $N(\varepsilon)$ is the regularization stability operator. The error $\|\boldsymbol{K}_h\varepsilon - u_{\delta}\|$ is a convex function, and the regularization stabilization operator takes different functions to form different solutions. Modern optimization inversion methods adopt smoothing functions based on norms [19], i.e. regularization solutions add filtering factors on the basis of least-squares methods or filtering high frequency components to achieve regularization.

However, there are many induced Dirac singularities in the aero-engine lubricant wear debris capacitance array. When the high frequency component is filtered out, the relative difference of energy of the multi-dirac function is less than the convex functional energy, and the boundary information of the lubricant abrasive metal is lost. Subsequently, the optimization method is not suitable for the new application of Multi-induced Dirac parameter reconstruction. The contrast changes sharply near the discontinuous boundary of metal abrasive particles and the solution is unstable [20].

2. Sensitivity of the Capacitance Array

As the size of wear debris decreases, the position between the wear debris and the plate recedes from the center of the plate gap, the distance between the abrasive particles and the plate increases, and the detection of the response signal decreases. The relationships between the detection response and the parameters of the wear debris size, the distance between wear debris, and the plate gap are all approximately linear, and the relationship between the detection response and the distance between the wear debris and plate is approximately exponential. The model is consistent.

The sensitivity distribution of wear debris is described by the following equations:

$$S(a_r)_{j,k} = 2\pi {\varepsilon_{oil}}^2 (a_r)^2 (\phi_r)^2 \varsigma_r. \quad (15)$$

$$S(\theta_r)_{j,k} = \varepsilon_{oil} \sum_{r=1}^{r=m} [\mathbf{T}_{\theta_r} \left(\frac{\partial \phi_r}{\partial \theta_r}, \frac{\partial \left(\frac{\phi_r}{\phi_t}\right)}{\partial \theta_r}, \frac{\partial R_{rt}}{\partial \theta_r} \right) (a_r)^2]. \quad (16)$$

$$S(\rho_r)_{j,k} = \varepsilon_{oil} \sum_{r=1}^{r=m} [\mathbf{T}_{\rho_r} \left(\frac{\partial \phi_r}{\partial \rho_r}, \frac{\partial \left(\frac{\phi_r}{\phi_t}\right)}{\partial \rho_r}, \frac{\partial R_{rt}}{\partial \rho_r} \right) (a_r)^2]. \quad (17)$$

In the equation, $S(a_r)_{j,k}$ is the size sensitivity of wear debris, $S(\theta_r)_{j,k}$ and$S(\rho_r)_{j,k}$ are the sensitivity of wear debris varying with angle and position. They are positively correlated with the square of the scale a_r of the wear debris themselves, with the induced field distribution ς_r and the field gradient projection $\mathbf{T}_r$ ($\mathbf{T}_{\theta_r}$ and $\mathbf{T}_{\rho_r}$ of the wear debris, which are polar angle and polar diameter variable projections, respectively). The sensitivity distribution is correlated with the unknown quantity, so the linear combination of the sensitivity distribution of the

single unknown quantity of the wear debris cannot be used to directly represent the position of the multi-wear debris. The field gradient projection $\mathbf{T}_r \geq 0$ is only related to the induced field when the excitation field distribution is uniform. That is to say, the conformal transformation of the excitation mapping the field distribution to the uniform field distribution is the projection operator of a single wear debris.
